# Arabidopsis Ubiquitin E3 Ligase AtCHYR1 Promotes ROS Production in Plant Responses to Sugar Availability

**DOI:** 10.3390/plants14172617

**Published:** 2025-08-22

**Authors:** Shuangcheng Ding, Yuxin Xue, Yulu Teng, Simin Qin, Hongwei Wang

**Affiliations:** 1 MARA Key Laboratory of Sustainable Crop Production in the Middle Reaches of the Yangtze River (Co-Construction by Ministry and Province), Yangtze University, Jingzhou 434025, China; shchding@yangtzeu.edu.cn; 2 Agricultural College, Yangtze University, Jingzhou 434025, China; 15872743050@163.com (Y.X.); 19846968186@163.com (Y.T.); 19071388367@163.com (S.Q.)

**Keywords:** AtCHYR1, Glc signaling, germination, root growth, ROS

## Abstract

Glucose functions as both an essential energy source and a critical signaling molecule, playing pivotal roles in regulating plant growth, development, and stress responses. Here, we report that *AtCHYR1*, a previously characterized RING-type ubiquitin E3 ligase involved in drought tolerance, also participates in glucose signaling. Exposure to high glucose levels significantly inhibits *AtCHYR1* expression, particularly in root tips, while low glucose conditions, such as osmotic stress, sugar starvation, and dark conditions, induce its expression. Importantly, analysis of *chyr1* mutants and plants overexpressing *AtCHYR1* revealed that *AtCHYR1* positively regulated the high glucose-mediated inhibition of germination and root growth, as well as starvation-induced growth retardation, through enhanced reactive oxygen species (ROS) accumulation in root tips. Additionally, transcriptional levels of glucose-activated pathogenesis-related (PR) and defense-related genes were reduced, while hypoxia-associated and ROS-inducing genes were significantly upregulated in *AtCHYR1*-overexpressing plants. Collectively, our findings provide novel insights into the role of *AtCHYR1* in plant responses to fluctuating sugar availability and its control of ROS homeostasis during seed germination and plant growth.

## 1. Introduction

The sugar produced during photosynthesis not only serves as a substrate for carbon and energy metabolism but also functions as a hormone-like molecule [1]. Throughout their life cycles, plants encounter various environmental stresses, including nutrient deficiency, extreme temperatures, drought, and high salinity. These stresses typically cause constant fluctuations in endogenous sugar levels by affecting photosynthesis and respiration [2]. Plants can sense sugar levels through specialized sensors, triggering a cascade of signal transduction processes that in turn induce changes in gene expression and protein modification [3]. Arabidopsis hexokinase 1 (AtHXK1) was the first plant intracellular glucose sensor identified in the glucose (Glc) signaling network, which is capable of sensing Glc and catalyzing the phosphorylation of Glc to glucose-6-phosphate in the initial step of glycolysis [4]. In the AtHXK1-dependent pathway, photosynthetic gene expression is correlated with the signaling function mediated by AtHXK1, whereas in the glycolysis-dependent pathway, the catalytic activity of AtHXK1 is required to regulate the expression of the pathogenesis-related (PR) genes [5,6]. In addition to being directly perceived by sensors, Glc signals are also indirectly induced by energy and metabolite sensors. Two energy sensors, AtKIN10/SnRK1 (sucrose-non-fermentation-related protein kinase1) and TOR-kinase (target of rapamycin kinase), are located downstream from sugar perception; they repress and activate growth under conditions of low and high energy levels, respectively [7,8]. In plants, SnRK1 plays a particularly important role in nutrient sensing and stress responses, coordinating plant growth and development with environmental conditions [8].

Glucose (Glc) is the most extensively studied hexose, playing a critical role in seed germination, root growth, and stress responses [1,9]. When grown on medium containing high exogenous Glc (e.g., 6%, approximately 330 mM), early germination and growth of Arabidopsis seedlings are hindered. Specifically, the radicle fails to break through the seed coat during germination, cotyledons cannot turn green quickly after germination, and hypocotyl and root elongation are inhibited. In root development, 1–3% Glc promotes increases in root length and lateral root number, whereas 5% Glc leads to a reduction in both parameters [10]. Extensive characterization of mutants has revealed that Glc signaling is closely associated with other signal pathways, including those mediated by hormones, stresses, and nutrients. Glc delays germination by activating the abscisic acid (ABA) signaling pathway and repressing the gibberellic acid signaling pathway [11]. Roots are not only crucial for plants to absorb water and nutrients from the soil but also serve as vital systems that help them adapt to the constantly changing environment [12]. Several factors determine optimal root growth and architecture, including hormonal levels (e.g., auxin) and nutrient availability (e.g., glucose). It has been reported that high concentrations of Glc reduce the size of the root meristem zone by repressing PIN1 accumulation, thereby decreasing auxin levels [13].

Reactive oxygen species (ROS), such as H_2_O_2_ and superoxide, serve as additional key signaling molecules regulating seed germination and root meristem activity in plants [14,15]. In particular, reduced efficiency of the mitochondrial electron transport chain, leading to superoxide radical formation, and osmotic stress-induced membrane disruptions that compromise ion homeostasis both contribute to ROS production [16,17]. A physiologically permissible level of ROS is required for seed germination and dormancy regulation [18]. In *Zinnia elegans* seeds, ROS also promote germination through the oxidation of germination inhibitors [19]. In contrast, excessive ROS interact with proteins, lipids, and DNA, oxidizing and modifying these components, impairing their normal function, damaging cell membranes, and ultimately leading to seed aging [20]. Additionally, excessive ROS have been reported to impair pollen germination and tube growth, and cause the retardation of root growth and programmed cell death (PCD) [21,22]. Although several studies have demonstrated that glucose promotes ROS production, the mechanism by which ROS regulate glucose-mediated root growth remains largely unknown and requires further investigation.

Post-translational modification by ubiquitination is a key regulatory mechanism in plant hormone signaling pathways [23]. This process is sequentially mediated by three enzymatic components: ubiquitin-activating enzyme (E1), ubiquitin-conjugating enzyme (E2), and ubiquitin ligase (E3) [24]. Within the E3 ligase family, RING-type proteins have garnered significant attention for their roles in ABA signaling, with several examples demonstrating their involvement in receptor degradation. For instance, RSL1 mediates the ubiquitination and subsequent degradation of ABA receptors PYR1 and PYL4 [25], while KEG promotes the degradation of ABI5, ABF1, and ABF3 via the 26S proteasome [26]. A special group of RING proteins are designated as CHYRs (CHY ZINC FINGER AND RING PROTEIN) protein due to their conserved N-terminal CxHYxR motif, referred to as the CHY zinc finger domain. Extensive studies have shown that *CHYRs* are involved in diverse abiotic stresses [27,28,29]. In *Arabidopsis thaliana*, there are seven *AtCHYR* genes. AtCHYR1 is phosphorylated by SNF1-RELATED PROTEIN KINASE 2.6 (SnRK2.6), which promotes ABA-induced stomatal closure, ROS production, and enhanced plant drought tolerance [30]; it also ubiquitinates phosphorylated WRKY DNA-BINDING PROTEIN 70 (WRKY70) to regulates the growth–immune balance [31]. AtCHYR2 (also known as BTS LIKE1, BTSL1), AtCHYR3 (BTSL2), and AtCHYR4 (also named as BRUTUS, BTS) are all involved in the Fe^2+^ deficiency response by mediating the ubiquitination and degradation of bHLH (basic helix-loop-helix) transcription factors [32,33]. AtCHYR6/MIEL1 (MYB30-Interacting E3 Ligase1) participates in ABA signaling, hormone metabolism, root development, and cuticle formation through the ubiquitination of MYB (myeloblastosis) transcription factors [34,35]. Thus, AtCHYR proteins target distinct substrate proteins for degradation to regulate diverse developmental processes and responses to abiotic/biotic-stresses.

Beyond ABA signaling, RING-type E3 ligases are increasingly recognized for their roles in sugar signaling. Examples include SIS3, which positively regulates sugar signaling during early seedling development [36], and ATL15 and ATL8, which modulate plant growth in response to sugar [37,38]. Recently, AtCHYR2 has been suggested to function as a positive regulator of glucose response [39]. However, the biochemical and physiological functions of many RING-type E3 ligases remain largely unexplored, representing a critical gap in our understanding of sugar signaling. In this study, we found that AtCHYR1, a previously characterized RING-type ubiquitin E3 ligase involved in drought tolerance, also participates in glucose signaling. While *AtCHYR1* was previously identified as a gene inducible by ABA and drought, we now show that its expression is repressed in response to various exogenous sugar treatments. Seed germination and primary roots in *chyr1-2* and *chyr1-3* mutants were both insensitive to high glucose and low glucose. Furthermore, these mutants exhibited reduced root ROS levels under varying sugar availability, suggesting that AtCHYR1 positively functions in the glucose responses by modulating ROS production. RNA-sequencing and qRT-PCR analyses revealed that *AtCHYR1* regulates the expression of a large set of genes in response to glucose, including activating hypoxia response genes and suppressing defense response genes, likely contributing to the regulation of cellular ROS levels. The findings presented in this study illuminate the important regulatory role of *AtCHYR1* in plant growth and development, and enhance our understanding of the mechanisms underlying plant sugar signaling.

## 2. Results

### 2.1. Expression of AtCHYR1 Is Repressed by High Concentration of Exogenous Sugar

**Figure 1:**
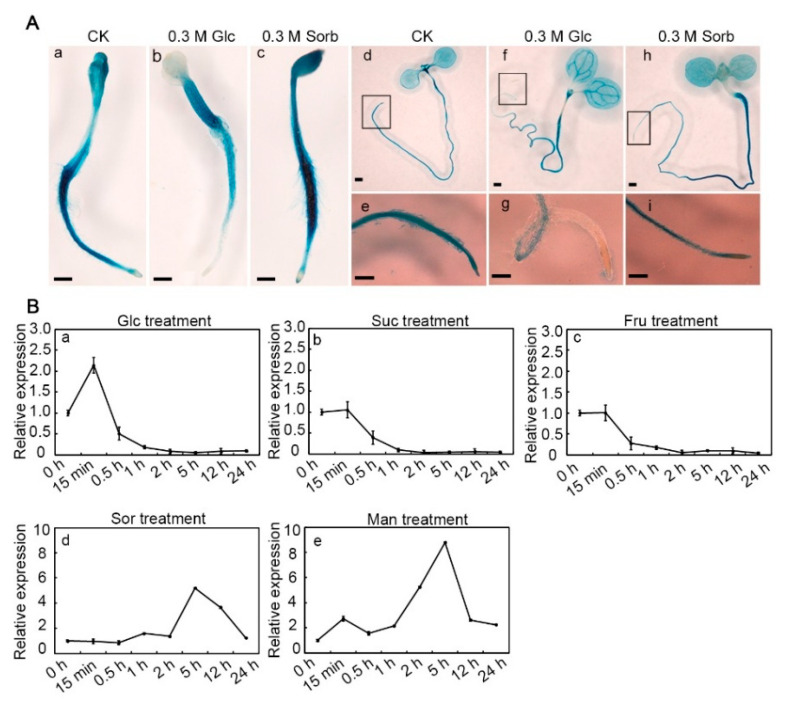
Analysis of *AtCHYR1* expression in response to various exogenous sugar treatments. (**A**) GUS staining of *proAtCHYR1:GUS* transgenic Arabidopsis T_3_ plants. Two-day-old seedlings were grown on MS medium (CK) (**a**), MS with application of 0.3 M of glucose (Glc) (**b**) or 0.3 M of sorbitol (Sorb) (**c**). Seven-day-old seedlings were treated with 0.3 M of glucose (Glc) (**f**,**g**) or 0.3 M of sorbitol (Sorb) (**h**,**i**), or without (CK) (**d**,**e**) for 10 h. Scale bar = 500 μm. (**e**,**g**,**i**) are the magnified views of the black-framed root tip regions in figures (**d**,**f**,**h**), respectively. (**B**) qRT-PCR analysis of *AtCHYR1* expression in response to 0.3 M of glucose (Glc) (**a**), sucrose (Suc) (**b**), fructose (Fru) (**c**), sorbitol (Sor) (**d**), or mannitol (Man) (**e**) for 24 h. Data represent the mean ± SD from three independent experiments, each with three technical replicates. Values were normalized using the Arabidopsis *18S rRNA* gene.

To investigate whether *AtCHYR1* expression is Glc-responsive, we analyzed histochemical GUS (β-Glucuronidase) staining of *proAtCHYR1:GUS* transgenic seedlings under high-glucose treatment. In 2-day-old and/or 7-day-old *proAtCHYR1:GUS* seedlings grown on Murashige and Skoog (MS) medium, GUS staining was detectable in the cotyledons, hypocotyl, root, and the shoot apical meristem (Figure 1A(a,d)). When 0.3 M of glucose (instead of sorbitol, a non-metabolizable sugar serving as the osmotic control) was added to the MS medium, a notable reduction in the GUS staining signal was detected in both cotyledons and roots (Figure 1A(b,c)). Similarly, when 7-day-old seedlings were treated with 0.3 M of glucose for 10 h, GUS staining exhibited relatively lower expression in the root tip (Figure 1A(d–g)). In contrast, under 0.3 M of sorbitol treatment for 10 h, GUS staining was significantly induced and localized to the vascular tissues of the root mature zone. Results from GUS activity assays indicated that *AtCHYR1* is likely repressed by high exogenous glucose (Figure 1A(h,i)).

Next, to verify whether sugars repress *AtCHYR1* expression, three-week-old WT seedlings were subjected to different exogenous sugar treatments, and expression was analyzed by quantitative real-time PCR (qPCR). As shown in Figure 1B(a), the time-course expression profile of the *AtCHTR1* gene revealed a 2-fold induction within 15 min of glucose treatment, followed by continuous and significant downregulation. The glucose-induced reduction in *AtCHYR1* expression was consistent with the GUS staining results. Furthermore, a similar trend was observed: *AtCHYR1* expression was also dramatically repressed after 15 min of treatment and maintained extremely low expression levels when responding to high exogenous sucrose or fructose (Figure 1B(b,c)). Interestingly, when exposed to equimolar concentrations of sorbitol or mannitol (both non-metabolizable sugar alcohols serving as controls), *AtCHYR1* expression exhibited peak inductions of ~6-fold and ~9-fold at 5 h, respectively (Figure 1B(d,e)). Here, osmotic stress caused by high concentrations of sorbitol or mannitol may mimic drought stress, thereby inducing *AtCHYR1* expression, which is consistent with previous findings [30]. Taken together, these results demonstrate that *AtCHYR1* is induced by osmotic stress but repressed by high concentrations of exogenous sugars.

### 2.2. AtCHYR1 Positively Promotes High Glc-Mediated Inhibition in Seed Germination and Post-Germination Growth

**Figure 2:**
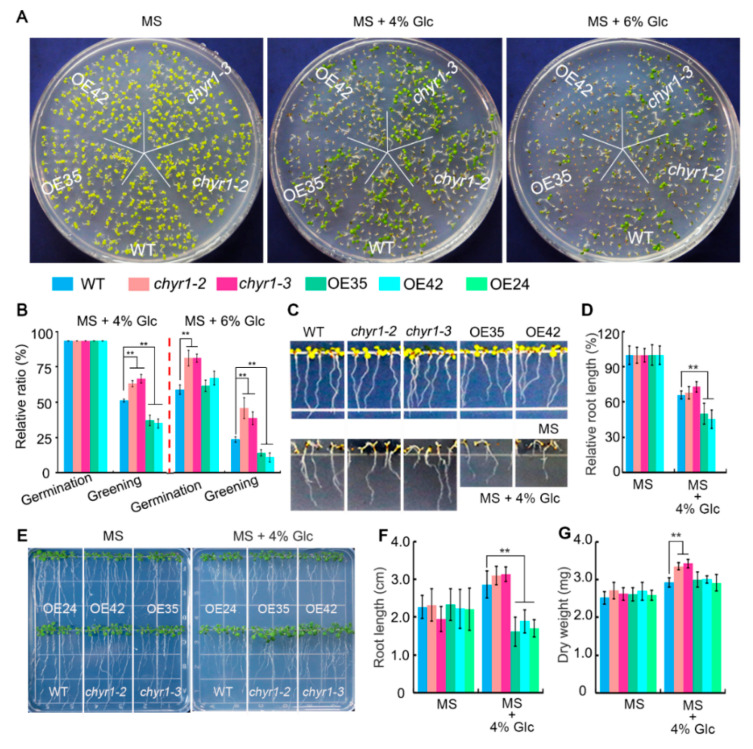
Phenotypes analysis of WT, *chyr1-2*, *chyr1-3*, OE35, and OE42 in response to high glucose during germination and post-germinative growth. (**A**) Representative images of seed germination on MS medium supplemented with 0%, 4%, or 6% glucose (Glc) after 10 days. (**B**) Rates of germination and cotyledon greening under 4% and 6% Glc. (**C**,**D**) Phenotypes of seedling (**C**) and primary root length (**D**) when germinated and grown under MS medium with 4% Glc for 7 days. (**E**–**G**) Visual comparison of root growth, primary root length (**E**), and the whole plant dry weight (**F**) of seedlings under 4% Glc treatments. Seedlings were grown on 1/2 MS medium for 4 days and then transferred to MS containing 4% Glc for another 7 days. Data are presented as mean ± SD (n = 3). Asterisks indicate significant differences compared to wild-type (** *p* ≤ 0.01; Student’s *t*-test).

Given that *AtCHYR1* expression is downregulated by sugars, we further analyzed the function of *AtCHYR1* in plant responses to sugar. WT, *chyr1* mutants (*chyr1-2*, *chyr1-3*), and *AtCHYR1* overexpressing lines (OE35, OE42) were grown on MS medium supplemented with different concentrations of exogenous glucose for 10 days (Figure 2A). When sown on MS medium containing 4% glucose, no difference in germination rates were observed among the genotypes (Figure 2B). However, *AtCHYR1*-overexpression seedlings exhibited a lower cotyledon greening rate compared with the WT plants and *chyr1* mutants. In the presence of 6% Glc, both germination and cotyledon greening rates were higher in *chyr1* mutants. In contrast, *AtCHYR1*-overexpressing plants displayed more severe post-germinative growth arrest, with only ~10% of their cotyledons expanding and developing to the four-leaf stage (Figure 2B). These results demonstrated that the loss of *AtCHYR1* reduces sensitivity to glucose-induced inhibition of seed germination, thereby alleviating glucose-mediated post-germinative arrest. Conversely, *AtCHYR1* overexpression enhances glucose-induced inhibition of seed germination, impairs cotyledon greening and expansion, and retards true leaf formation.

Furthermore, we observed that *AtCHYR1*-overexpressing lines displayed a hypersensitivity phenotype when germinated and grown in the presence of 4% Glc (Figure 2C,D). To verify that the Glc-induced reduction in root growth of *AtCHYR1*-overexpressing seedlings was not due to impaired seed germination, 4-day-old seedlings of wild-type (WT), *chyr1* mutant, and *AtCHYR1*-overexpressing lines were transferred to media supplemented with 4% Glc and cultured for 7 days (Figure 2E). Compared with WT, primary root growth of *AtCHYR1*-overexpressing seedlings was significantly inhibited on 4% Glc medium (Figure 2F). Conversely, *chyr1* mutants showed insensitivity to high Glc-mediated suppression of shoot and root growth, as evidenced by their higher dry weight compared with WT plants (Figure 2G). These results suggest that AtCHYR1 positively regulates glucose-mediated inhibition of germination, as well as post-germinative growth arrest, including cotyledon expansion, leaf formation, and root growth.

### 2.3. AtCHYR1 Is a Glc Starvation-Response Gene and Aggravates Plant Starvation Response

**Figure 3:**
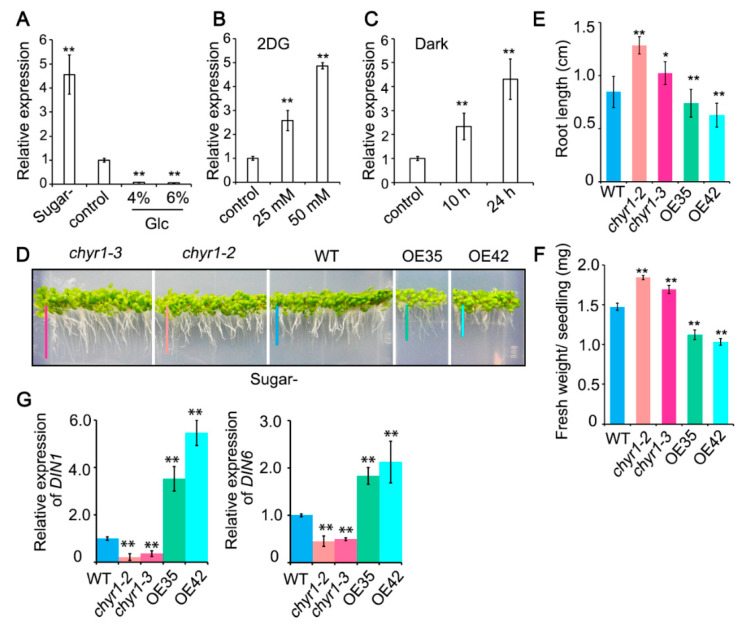
*AtCHYR1* expression and phenotypic analysis under sugar starvation. (**A**) qRT-PCR analysis of *AtCHYR1* expression in WT seedlings grown on MS plates with different concentrations of glucose for 7 days. “Sugar-” refers to the absence of sugar in the medium. (**B**) qRT-PCR analysis of *AtCHYR1* expression in WT seedlings treated by different concentrations of 2-Deoxy-D-Glucose (2DG) for 24 h. (**C**) qRT-PCR analysis of *AtCHYR1* expression in WT seedlings treated by darkness. (**D**–**F**) Phenotypes of primary root length (**E**) and fresh weight (**F**) of *chyr1* mutants (*chyr1-2*, *chyr1-3*) and *AtCHYR1*-overexpression lines (OE35, OE42) grown on sugar-free medium for 10 days. The lines of different colors in the (**D**) are intended to intuitively indicate the length of the roots. (**G**) Expression profiles of *DIN1* and *DIN6* in *chyr1* mutants and *AtCHYR1*-overexpression seedlings. For qRT-PCR analysis, *18S rRNA* was used as an endogenous control. Error bars represent standard deviation (SD) from three independent biological replicates. Asterisks indicate statistically significant differences compared to the WT (** *p* ≤ 0.01, * *p* ≤ 0.05; Student’s *t*-test).

High concentrations of sugar lead to energy excess in plant cells, whereas ABA, drought, or osmotic stress may reduce sugar availability in plants. Given the close links between sugar/energy availability and abiotic stress, we further examined *AtCHYR1* expression in response to sugar-limited conditions to investigate whether *AtCHYR1* plays a role in sugar starvation responses. We firstly performed a dose-dependent Glc treatment assay using 5-day-old wide-type seedlings. The results clearly showed that *AtCHYR1* expression was gradually repressed as Glc concentrations increased (Figure 3A). When treated with 2-deoxy-D-glucose (2DG), a compound that causes energy depletion by blocking glycolysis [40], *AtCHYR1* expression increased in a dose-dependent manner in response to 2DG treatment (Figure 3B). Moreover, *AtCHYR1* was found to be positively regulated by dark treatment (Figure 3C). These results confirmed that the *AtCHYR1* level is induced by energy starvation. Interestingly, when WT, *chyr1* mutants, and *AtCHYR1*-overexpressing seedlings were germinated and grown on a sugar-free medium, the mutants exhibited better growth than both the overexpressing lines and the WT (Figure 3D). Additionally, *AtCHYR1*-overexpressing lines showed reductions in both primary root length and fresh weight compared with WT, while *chyr1* mutants displayed notable increases in these two growth parameters (Figure 3E,F). Furthermore, we measured the relative expression levels of starvation-inducible genes *DIN1* and *DIN6*. Compared with WT, the expression levels of these genes were significantly reduced in mutants but increased in overexpressing lines (Figure 3G). Collectively, these results indicate that *AtCHYR1* is a starvation-response gene that promotes starvation responses, which curtail primary root growth and hinder shoot growth under severe sugar limitation.

### 2.4. AtCHYR1 Enhances ROS Accumulation in Roots Under High-Glucose Conditions and Sugar-Starvation

**Figure 4:**
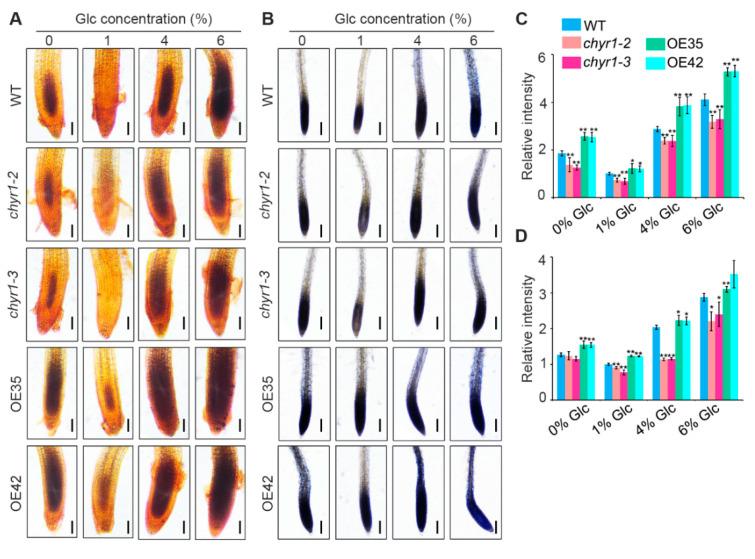
ROS accumulation in root tips under varying glucose concentrations. (**A**) Root tips were subsequently stained with DAB to visualize H_2_O_2_. Bar = 50 µM. (**B**) Root tips were subsequently stained with NBT to visualize O_2_^•−^. Bar = 50 µM. (**C**) Relative quantification based on the brown intensity of DAB staining (**A**). (**D**) Relative quantification based on the blue intensity of NBT staining (**B**). Five-day-old seedlings were grown vertically on 1/2 MS agar plates supplemented with different concentrations (0%, 1%, 4%, and 6%) of exogenous glucose (Glc) for 24 h. Data are expressed as means ± SD from three independent biological replicates. Significant differences compared to WT are indicated by asterisks (* *p* ≤ 0.05; ** *p* ≤ 0.01; Student’s *t*-test).

ROS play a key regulatory role in the germination program under high exogenous sugar conditions [21]. Therefore, in this study, we examined ROS accumulation in WT, *chyr1* mutants, and *AtCHYR1*-overexpressing seedlings under varying sugar concentrations. DAB (3,3′-diaminobenzidine) and NBT (nitroblue tetrazolium) staining were used to detect H_2_O_2_ and O_2_^•−^ in situ, respectively. Plants grown under normal sugar conditions (1/2 MS medium supplemented with 1% glucose) exhibited relatively low ROS levels. In contrast, pronounced accumulation of H_2_O_2_ (Figure 4A) and O_2_^•−^ (Figure 4B) was detected in the meristematic zones of the primary root under high exogenous glucose conditions. We found that ROS concentrations in the *chyr1* mutants were consistently lower than those in WT across varying exogenous glucose treatments, whereas ROS levels in OE35 and OE42 lines were higher than those in WT (Figure 4). Additionally, ROS content analysis revealed that *AtCHYR1*-overexpressing lines had significantly higher ROS levels than WT even under sugar-free treatment (Figure 4). These findings suggest that *AtCHYR1* enhances root ROS accumulation under both exogenous sugar treatments and sugar-starvation conditions.

### 2.5. Transcriptomic Analysis Reveals That Glc-Inducible Genes Are Regulated in AtCHYR1-Overexpressing Plants

**Figure 5:**
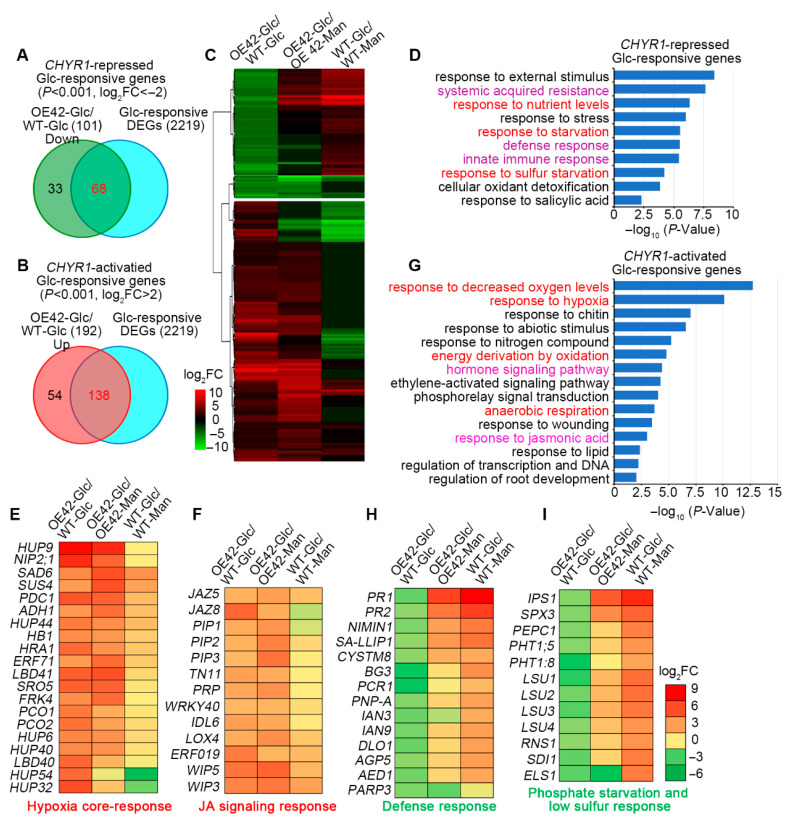
Analysis of *AtCHYR1*-regulated and glucose-responsive genes. (**A**,**B**) Venn diagrams illustrating the overlap between *AtCHYR1*-repressed (**A**) and *AtCHYR1*-activated (**B**) glucose-responsive genes. (**C**) *K*-means clustering of 206 *AtCHYR1*-regulated and glucose-responsive genes, revealing expression patterns. (**D**) GO enrichment analysis of 68 *AtCHYR1*-repressed and glucose-responsive genes, highlighting enriched biological processes. (**E**,**F**) Heatmaps showing expression profiles of genes related to hypoxia core-response (**E**) and jasmonic acid (JA) signaling response (**F**). (**G**) GO enrichment analysis of 138 *AtCHYR1*-activated and glucose-responsive genes, identifying enriched biological functions. (**H**,**I**) Heatmaps displaying expression profiles of genes involved in defense response (**H**), and phosphate starvation and low sulfur response (**I**).

To gain deeper insights into the functions of *AtCHYR1* in Glc response, we established four experimental groups: OE42 treated with Glc (OE42-Glc), wild type treated with Glc (WT-Glc), OE42 treated with mannitol (OE42-Man), and wild type treated with mannitol (WT-Man). As shown in Table S1, after excluding differential expression genes (DEGs) from the comparisons of WT-Glc vs. WT-Man or OE42-Glc vs. OE42-Man, we identified 2219 glucose-regulated DEGs by comparing OE42-Glc against WT-Glc. Using a significance threshold of *p* ≤ 0.001 and |log_2_FC| ≥ 2, we designated 68 genes as *AtCHYR1*-activated Glc-responsive genes and 138 genes as *AtCHYR1*-repressed Glc-responsive genes (Figure 5A,B). Cluster analysis clearly divided these 206 DEGs into two distinct groups (Figure 5C, Table S1).

Gene ontology (GO) analysis identified ten significantly enriched biological processes among the 68 *AtCHYR1*-repressed and Glc-responsive genes (Figure 5D). These processes included the response to stimulus, stress, salicylic acid, systemic acquired resistance (SAR), defense response, nutrient level changes, starvation, and cellular oxidant detoxification. Notably, key defense response marker genes, such as *PR1* (*pathogenesis-related protein 1*), *PR2,* and *PNP-A* (*plant natriuretic peptides A*), were enriched in this group (Figure 5E). Furthermore, 12 marker genes involved in phosphate starvation responses and low sulfur responses were also enriched within this cohort (Figure 5F). Transcriptomic analysis revealed that genes related to defense response and nutrient signaling pathways are activated under glucose induction in WT plants, whereas these pathways were significantly inhibited in the OE42 transgenic line. This inhibition may prevent OE plants from effectively coping with high-glucose-induced metabolic disorders, thereby affecting the energy supply for seed germination and disrupting the maintenance of root growth homeostasis. These findings suggest a critical role for *AtCHYR1* in modulating crosstalk between nutrient and defense pathways under Glc-induced metabolic stress.

Among the 138 *AtCHYR1*-activated and Glc-responsive genes, GO enrichment analysis identified 15 enriched terms (Figure 5G). These genes were primarily associated with responses to decreased oxygen levels, hypoxia, anaerobic respiration, energy derivation, hormone-mediated signaling pathways, the regulation of transcription, response to nitrogen compounds, wounding, salicylic acid, and the regulation of root development (Figure 5G). Notably, four GO terms, the response to decreased oxygen levels, hypoxia, anaerobic respiration, and energy derivation, are closely linked to energy status and production. Surprisingly, dozens of core hypoxia-responsive genes, including *HB1*, *ADH1* (*alcohol dehydrogenase 1*), *PDC1* (*pyruvate decarboxylase 1*), *PCO1* (*plant cysteine oxidase 1*), and *PCO2*, displayed significantly higher expression levels in OE transgenic lines than in WT in response to glucose (Figure 5E). The elevated expression of *ADH1* and *PDC1,* genes encoding key limiting enzymes in the anaerobic respiration pathway, is likely to enhance pyruvate-to-ethanol conversion, and promote mitochondrial ROS generation. Within the GO term for hormone signaling pathways, 13 jasmonic acid (JA) signaling marker genes were identified (Figure 5F). Notably, JA signaling repressors of the JAZ (jasmonate ZIM domain protein) family, including JAZ5 and JAZ8, which negatively regulate JA-mediated root growth processes, were not significantly activated in Glc-treated WT seedlings but were clearly induced in OE42 plants. Collectively, the synergistic activation of hypoxia responses and JA signaling may exacerbate the sensitivity of seed germination and root growth to high glucose by aggravating oxidative damage and disrupting root development regulation.

**Figure 6:**
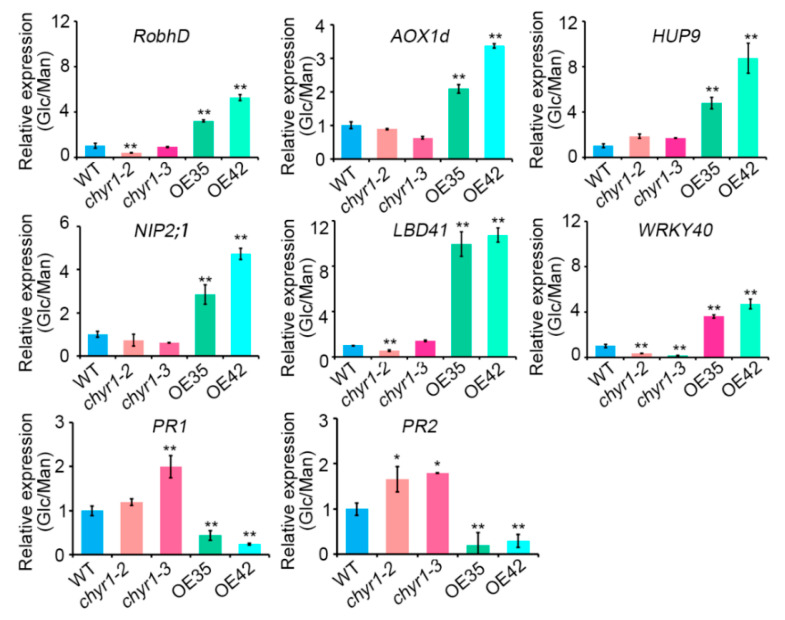
*AtCHYR1* regulates the expression level of marker genes involved in Glc signaling response. qRT-PCR analysis was performed with 7-day-old seedlings of WT, *chyr1-2*, *chyr1-3*, and *35S:AtCHYR1* lines, which were treated with 0.3 M of glucose or mannitol for 24 h. *18S rRNA* transcripts were used as an internal control. Significant differences compared to the wild type (WT) are indicated by asterisks (* *p* ≤ 0.05; ** *p* ≤ 0.01; Student’s *t*-test).

The expression of hypoxia- or defense-inducible genes was further analyzed in WT, *chyr1-2*, *chyr1-3*, and *AtCHYR1*-overexpression transgenic plants by qRT-PCR (Figure 6). Under high-glucose treatment, *RobhD*, *AOX1d*, *HUP9,* and *NIP2;1*, genes which were responsible for ROS homeostasis and the response to low oxygen stress, were induced to a greater extent in OE35 and OE42 compared to their respective mannitol-treated controls, whereas their induction was reduced in *chyr1-2* and *chyr1-3* mutants. Similar expression patterns were observed for *LBD40* and *WRKY40*, which act as central transcriptional repressors in seed germination and root growth [41,42]. In contrast, the expression of pathogenesis-related genes *PR1* and *PR2* was significantly downregulated in OE35 and OE42 lines under high-glucose treatment compared to their mannitol-treated controls, relative to WT plants, while showing slight upregulation in the mutants. Collectively, these findings suggest that *AtCHYR1* specifically activates the hypoxia response while suppressing the glucose-induced defense pathway, a regulatory mechanism that is crucial for balancing plant growth and defense.

## 3. Discussion

Glucose functions both as a nutrient and a signaling molecule, participating in various cellular processes including embryogenesis and germination [9]. Low concentrations of glucose (0.5–2% *w*/*v*) are known to delay germination but have a minor impact on subsequent development [43]. In contrast, moderate glucose concentrations (2–6% *w*/*v*) severely alter seedling growth, manifesting as halted seed mobilization, slowed chloroplast development, reduced cotyledon expansion, and a failure to develop true leaves and root systems. The underlying mechanism for this response remains unclear. Accumulating evidence suggests that glucose signaling interacts with multiple phytohormones to form a dynamic, integrated signaling network, facilitating adaptive growth, development, and stress responses in plants [4,44]. Although numerous key proteins in the glucose signaling pathway have been identified, the characterization of E3 ubiquitin ligases involved in sugar signal responses remains relatively limited. Previously, we demonstrated that AtCHYR1, a RING E3 ligase, is phosphorylated by SnRK2.6 to promote stomatal closure during drought stress responses [30]. In the present study, we found that high Glc-mediated inhibition of germination and primary root growth was significantly enhanced in *AtCHYR1*-overexpression plants, whereas *chyr1* mutants showed the attenuated inhibition of primary root growth (Figure 2). Notably, both high and low sugar levels induced ROS accumulation, and primary root ROS levels were strongly genotype-dependent. *chyr1* mutants exhibited reduced ROS accumulation in roots under high exogenous glucose, while *AtCHYR1*-overexpression lines consistently displayed higher ROS levels (Figure 4). Collectively, these results indicate that *AtCHYR1* positively regulates plant sugar signaling.

ROS are key signaling molecules in plant stress responses, and their excessive accumulation can cause oxidative damage [45]. E3 ubiquitin ligases have been shown to play crucial roles in regulating protein stability and activity, particularly in the context of oxidative stress and ROS metabolism. For example, OsPUB15, an E3 ubiquitin ligase, functions to reduce cellular oxidative stress during seedling establishment [46]; similarly, the E6AP E3 ubiquitin ligase regulates cellular response to oxidative stress [47]. Understanding these interactions may provide insights into cellular mechanisms underlying stress tolerance. In this study, the modulation of ROS levels by *AtCHYR1* is consistent with its involvement in both high exogenous Glc and sugar-starvation conditions. The lower ROS accumulation in mutants and the higher accumulation in overexpressing lines suggest that *AtCHYR1* normally promotes ROS production under varying sugar availability. This aligns with the known role of E3 ligases in regulating protein stability and activity, potentially by targeting ROS-generating enzymes for degradation [48,49].

It is well established that sugar sensors act as molecular hubs and integrators of metabolic and hormonal signals. Several key energy sensors have been identified in plants [9], which are activated or repressed in response to cellular sugar or energy levels [50,51,52]. From our analysis, we found that the cellular sugar and energy levels also serve as important regulators of *AtCHYR1* transcription. *AtCHYR1* expression was induced during energy starvation (e.g., 2DG and darkness treatments) (Figure 3B,C) but was repressed by exogenous metabolic sugars (Figure 2). In general, adverse environmental stresses such as drought can impair photosynthesis and/or respiration, leading to reduced ATP production and leaving cells in a low-energy state [7]. Combined with our previous finding that drought induces the upregulated expression of *AtCHYR1* [30], these results indicate that *AtCHYR1* expression is closely linked to intracellular energy status. Additionally, we observed that the inhibition of primary root and shoot growth under severe sugar limitation was aggravated in the *AtCHYR1*-overexpression line (Figure 3D–F). This suggests an interesting hypothesis: intracellular *AtCHYR1* levels may act as a signal of cellular energy status, with high *AtCHYR1* expression indicating that cells are coping with low energy levels.

Notably, transcriptomic analysis revealed that *AtCHYR1* overexpression activates the expression of numerous core hypoxia-responsive genes (Figure 5D,E), which likely enables plants to enhance carbohydrate catabolism to support growth. Simultaneously, OE plant-activated hypoxia-related genes play a critical role in promoting ROS production. Elevated expression of these hypoxia-responsive genes enhances anaerobic respiration, accelerating pyruvate-to-ethanol conversion. This metabolic shift induces NADH/NAD^+^ imbalance, disrupts electron transport chain function, and triggers mitochondrial ROS leakage, providing a mechanistic explanation for how *AtCHYR1* promotes ROS production in response to glucose signaling. Additionally, transcriptomic analysis showed that *AtCHYR1*-overexpression plants inhibit the activation of defense and nutrient signaling pathways, which are induced under high glucose in WT. The functional deficiency of these pathways likely weakens OE plants’ adaptability to metabolic disorders. The dampened defense signaling pathways in *AtCHYR1*-overexpression transgenic lines (Figure 5G) aligns with previous reports that AtCHYR1 ubiquitinates phosphorylated WRKY70 for degradation to balance immunity and growth [30]. Thus, AtCHYR1 likely plays a key role in coordinating energy allocation and defense activation under glucose stress.

## 4. Materials and Methods

### 4.1. Plant Materials and Growth Conditions

All Arabidopsis plants utilized in this study were generated from the Col-0 ecotype background. Two mutants [30], specifically *chyr1-2* and *chyr1-3*, along with three overexpressing lines of *AtCHYR1* (OE24, OE35, and OE42) [30], were employed in the research. Seeds were initially germinated on Murashige and Skoog (MS) medium supplemented with 3% sucrose and 0.6% agar (pH 5.8), after which they were cultivated in a growth chamber under conditions of 50% relative humidity at 22 °C, exposed to a photoperiod of 16 h of light followed by 8 h of darkness.

### 4.2. Germination Assay and the Response of the Root to Glc

To investigate the effects of Glc on seed germination, cotyledon greening, and post-germinative growth, seeds harvested and stored under identical conditions were sown on MS medium plates supplemented with 1% sucrose and varying concentrations of Glc. After ten days, the germination rate, the proportion of seeds with greened cotyledons, and the percentage of seedlings reaching the quad-leaf stage were quantified relative to the total number of germinated seeds.

For analyzing the impact of Glc on root growth, seedlings were either germinated and grown vertically on half-strength MS plates with or without 4% Glc for seven days, or initially grown on half-strength MS medium for four days before being transferred to half-strength plates containing 4% Glc or no Glc, followed by continued vertical growth for an additional seven days. All assays evaluating Glc-induced phenotypes were performed in triplicate experiments for each treatment.

### 4.3. Histochemical GUS Staining

To analyze GUS activity in response to Glc, 2-day-old *proAtCHYR1:GUS* transgenic lines were grown on MS medium supplemented with 1% sucrose, 0.3 M of Glc, or 0.3 M of sorbitol (as an osmotic control). Additionally, 5-day-old *proAtCHYR1:GUS* transgenic lines were subjected to treatment with either 0.3 M of Glc or 0.3 M of sorbitol (as an osmotic control) for 6 h. All the seedlings were then collected and processed for GUS staining.

The collected seedlings were briefly immersed in chilled 90% acetone prior to overnight incubation (~16 h) at 37 °C in a GUS staining solution, which contained 1 mM of X-GlcA, 2 mM of K_3_Fe(CN)_6_, 2 mM of K_4_Fe(CN)_6_, 10 mM of EDTA, and 0.1% (*v*/*v*) Triton X-100 in 100 mM of sodium phosphate buffer (pH 7.2). After staining, chlorophyll was removed by immersing the seedlings in 70% (*v*/*v*) ethanol. Stained seedlings were imaged using a stereoscope equipped with Nikon NIS Elements D software (version 3.7).

### 4.4. ROS Analyses

H_2_O_2_ and O_2_^−^ were detected via DAB and NBT, respectively. Five-day-old seedlings grown on 1/2 MS were transferred to 1/2 MS agar plates supplemented with different concentrations of exogenous glucose (0%, 1%, 4%, and 6%) and incubated for 24 h. For staining, roots were infiltrated with 10 mM of MES (pH 6.5) buffer containing 0.1% (*w*/*v*) DAB (for H_2_O_2_) or 50 mM of sodium phosphate buffer containing 0.05% (*w*/*v*) NBT (for O_2_^−^) and incubated in the dark for 8 h. Staining was terminated by immersing roots in boiling water for 20 min. After staining, roots were decolorized in 95% ethanol in a 95 °C water bath. Finally, images were captured using a confocal laser scanning microscope (Carl Zeiss LSM710, Jena, Germany).

ROS accumulation was quantified using Image J software (1.54 g). For each root, the region of interest (ROI) was defined as the primary root meristem zone (2–3 mm from the root tip). The integrated density (sum of pixel intensities) of the stained area within the ROI was measured, and the background intensity from unstained root regions was subtracted. At least 15 roots per genotype and treatment were analyzed, and relative ROS levels were calculated as the mean integrated density ± standard error.

### 4.5. RT-qPCR Analysis

To investigate the effects of sugar treatment on gene expression, 3-week-old seedlings were removed from MS medium plates and immersed in solutions containing 300 mM of Glc, 300 mM of sucrose, 300 mM of mannitol, or 300 mM of sorbitol. Total RNA was isolated using Trizol reagent (Biotopped, Beijing, China) and treated with RNase-free DNase I to eliminate residual genomic DNA contamination.

Two micrograms of total RNA were reverse-transcribed into cDNA using M-MLV Reverse Transcriptase according to the manufacturer’s instructions (Promega, Wuhan, China). Quantitative PCR (qPCR) was performed on the CFX96 real-time PCR detection system (Bio-Rad, Wuhan, China) with SYBR Green Master Mix (Takara, Wuhan, China). The relative expression of *AtCHYR1* was validated using *AtCHYR1*-specific primers [30], and *18S rRNA* was used as an internal reference gene for normalization.

### 4.6. RNA-Seq Data Analysis

For RNA sequencing, two-week-old seedlings from the *AtCHYR1*-overexpression line (OE42) and WT were treated with either 300 mM of Glc or mannitol for 24 h. Total RNA was isolated from these samples to construct cDNA libraries. Three biological replicates were prepared for each sample, resulting in a total of 12 libraries sequenced at the Beijing Genomics Institute (Wuhan, China) using the Illumina HiSeq X Ten system to generate 150 bp paired-end reads per library.

RNA-seq data analysis was conducted using STAR version 2.7.3a for alignment, featureCountsversion 2.0.1 for abundance estimation, and DESeq2 (version 1.30.1) for differential gene expression analysis [53,54,55]. Specifically, STAR aligned RNA-Seq reads to the Arabidopsis genome, while featureCounts estimated transcript abundances, and DESeq2 identified differentially expressed genes based on the following criteria: |log_2_ fold change| > 1; *p*-value < 0.05 [54,55,56].

Gene annotation files and GO terms were downloaded from The Arabidopsis Information Resource (TAIR) homepage (http://www.arabidopsis.org) (accessed on 22 August 2024). Clustering was applied to partition gene sets and comparisons into different clusters according to expression profiles using the ‘hclust’ function in R (version 4.3.3), with the distance set as Euclidean and the method set as Ward’s D. Log_2_ fold change values were used as input for clustering.

## 5. Conclusions

In summary, our study establishes a novel role for AtCHYR1 as a critical regulator in plant responses to sugar availability, where it modulates ROS homeostasis to control seed germination and seedling growth. Mechanistically, AtCHYR1 exerts its function through coordinated transcriptional regulation: it activates core hypoxia-responsive genes to facilitate energy derivation under metabolic stress and represses defense-related genes to fine-tune growth-defense trade-offs. These findings further solidify the emerging paradigm that E3 ubiquitin ligases serve as key molecular nodes integrating sugar signaling with stress response pathways. By demonstrating that AtCHYR1 acts as a molecular bridge linking sugar sensing, energy metabolism, and stress defense modulation, this study advances our understanding of the intricate signaling crosstalk between nutrient availability and stress adaptation in plants. Such insights contribute to the broader knowledge of plant metabolic stress responses and may inform strategies for enhancing crop resilience to nutrient fluctuations.

## Data Availability

All relevant data can be found within the manuscript. The RNA-seq data analyzed in this study have been submitted to NCBI with accession number PRJNA1260584.

## Supplementary Materials

The following supporting information can be downloaded at https://www.mdpi.com/article/10.3390/plants14172617/s1, Table S1: The Glc-responsive DEGs in *AtCHYR1* over-expression line (OE19); Table S2: Genes and primers used for verifying gene expression.
